# Supplementary material for: Statins and Colorectal Cancer Risk: A Population-Based Case-Control Study and Synthesis of the Epidemiological Evidence
Source: J Clin Med. 2022 Mar 10;11(6):1528. doi: 10.3390/jcm11061528 (PMC8953421; doi:10.3390/jcm11061528)
Supplement: Supplementary file 1 [file jcm-11-01528-s001.zip › jcm-1574521-supplementary.pdf]

## Supplementary Materials

### Figure S1. Vascular risk factors, comorbidity and comedication associated with current use of statins as compared to non-use, among controls

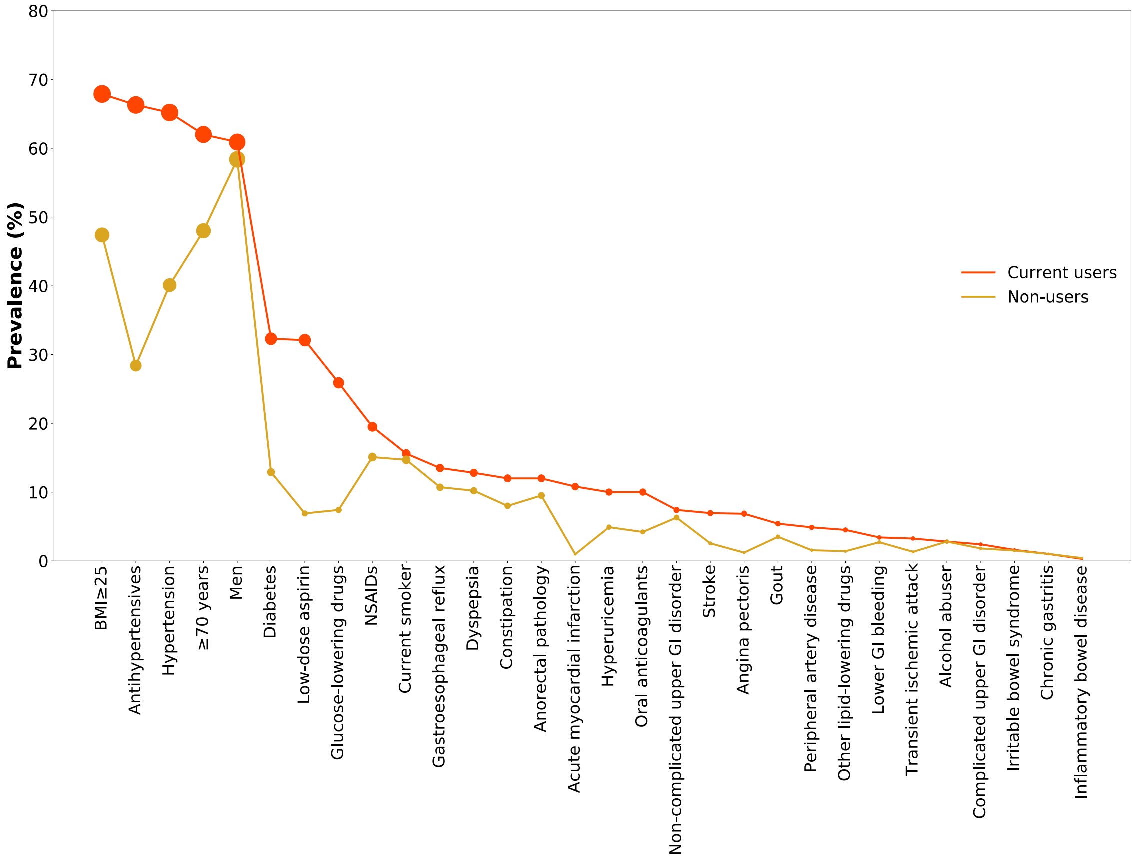


BMI: body mass index; NSAID: nonsteroidal anti-inflammatory drugs; GI: gastrointestinal

**Figure S2. Flow diagram for systematic review**

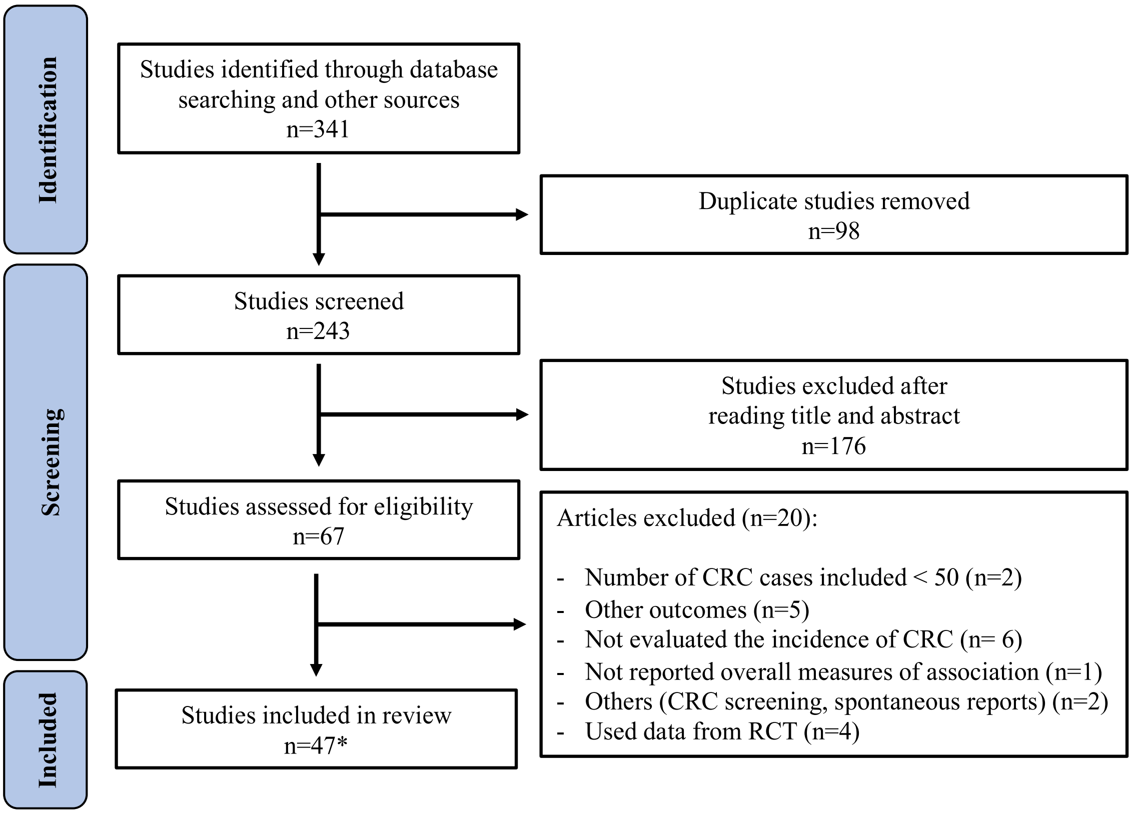


CRC: colorectal cancer; RCT: randomized clinical trials

*48 studies finally included in the meta-analysis: 47 selected after systematic review plus the present study.

**Table S1. Summary evidence from studies included in review**

**a) Case-control studies**

| Author (year, country) | Data source/ Study period | Exposure/ Active principles studied | Outcome/ Definition-confirmation | Definition of population | N | Cases | Site, sex | AOR (95% CI) | Matching/ Adjustment variables |
|---|---|---|---|---|---|---|---|---|---|
| **Blais *et al* (2000, Canada)** | Database (Régie de l'Assurance-Maladie du Québec), January 1, 1988-December 31,1994 | Prevalent statin users (at least 1 prescription). Reference category: bile acid-binding resins/ Simvastatin, Lovastatin, Pravastatin | Diagnosis of cancer/ ICD-9 (codes 153.0-154.9) | General population. Range age: ≥65 - ≥85 | 5962 | 56 | Colon, both sexes | 0.83 (0.37-1.89) | No/ 1,2,10, 16,57,58 |
| **Graaf *et al* (2004, Netherlands)** | Database (PHARMO), January 1, 1985-December 31, 1998 | Prevalent statin users (threshold of at least 6 months)/ Simvastatin, Atorvastatin, Fluvastatin, Cerivastatin, Pravastatin | First diagnosis of cancer/ ICD9-CM (codes 140 to 208) | Population with one or more prescriptions for cardiovascular drugs. Range age: ≤64 - ≥85 | 20105 | 292 | Colon, both sexes | 0.87 (0.48-1.57) | Yes/1-5,8-16 |
| | | | | | | 148 | Rectum, both sexes | 0.48 (0.16-1.48) | |
| **Kaye *et al* (2004, United Kingdom)** | Database (GPRD), 1990-2002 | Prevalent statin users (1 prescription within the year before cancer diagnosis, first prescription more than 1 year). Reference category: non-statin (fibrates, cholestyramine, colestipol, nicotinic acid, and acipimox). Hyperlipidemia not treated separate category/ Not defined | First diagnosis of cancer/ Not defined | Population with use of antihyperlipidemic drugs or diagnosis of untreated hyperlipidemia. Range age: 50-89 | 18088 | 329 | Colon, both sexes | 1.00 (0.60-1.70) | Yes/1-3,5,7, 17,18,28 |
| | | | | | | 183 | Rectum, both sexes | 1.60 (0.90-2.80) | |
| **Khurana *et al* (2004, United States)** | Database (VISN 16 VA), 1998-2004 | Prevalent statin users / Not defined | Diagnosis of cancer/ Not defined | General population. Mean age: 61.1 (SD ±14.4) | 534273 | 5339 | Colon, both sexes | 0.94 (0.89-1.00) | No/1,2,6,14, 17-19 |
| **Poynter *et al* (2005, Israel)** | Interview/ questionnaire, Database (Clalit Health Services), May 31, 1998- March 31, 2004 | Prevalent statin users (use at least for 5 years). Reference category: less than 5 years or no use/ Simvastatin, Pravastatin | Diagnosis of cancer/ Standardized pathological review | General population. Mean age cases: 69.9 (SD ±11.7) | 3968 | 1953 | Colorectal, both sexes | 0.53 (0.38-0.74) | Yes/1-3,6, 14,20-23 |

| Author (year, country) | Data source/ Study period | Exposure/ Active principles studied | Outcome/ Definition-confirmation | Definition of population | N | Cases | Site, sex | AOR (95% CI) | Matching/ Adjustment variables |
|---|---|---|---|---|---|---|---|---|---|
| **Rubin *et al* (2005, Denmark)** | Database (administrative claims), January 1, 2001-December 31, 2003 | Prevalent statin users (statin use measured during a 12-month period prior to diagnosis)/ Not defined | First diagnosis of cancer/ Colonoscopy, bowel surgery | Patients with inflammatory bowel disease (IBD-ulcerative colitis and/or Crohn's disease). Age ≥ 18 | 1536 | 364 | Colorectal, both sexes | 0.74 (0.52-1.05) | Yes/1,2,5,7, 14,24-28 |
| | | | | Patients without IBD. Age ≥ 18 years | 385704 | 18076 | Colorectal, both sexes | 0.92 (0.89-0.96) | |
| **Coogan *et al* (2007, United States)[a]** | Interview/ questionnaire, 1991-2005 | Prevalent statin users (use at least 4 times a week for at least 3 continuous months)/ Simvastatin, Lovastatin, Fluvastatin, Pravastatin | First diagnosis of cancer/ Not defined | General population. Range age: 40-79 | 8813 | 726 | Colorectal, both sexes | 0.80 (0.50-1.20) | No/1.1-3, 6, 14,17-19, 22,28,29 |
| | | | | | | 467 | Colon, both sexes | 0.70 (0.40-1.10) | |
| | | | | | | 259 | Rectum, both sexes | 1.10 (0.60-2.00) | |
| **Coogan *et al* (2007, United States)[b]** | Interview/ questionnaire, Database (Massachusetts Cancer Registry) January 1, 2001-November 30, 2004 | Prevalent statin users (use at least 3 times a week for at least 3 continuous months)/ Simvastatin, Lovastatin, Atorvastatin, Fluvastatin, Cerivastatin, Pravastatin, Rosuvastatin | First diagnosis of cancer/ Not defined | General population. Range age: 50-74 | 3618 | 1809 | Colorectal, both sexes | 0.92 (0.78-1.09) | Yes/1.1-3,6, 14, 17-19, 20,22,27,29-31 |
| | | | | | | 187 | Colorectal, women | 1.02 (0.78-1.32) | |
| | | | | | | 270 | Colorectal, men | 0.86 (0.68-1.08) | |
| | | | | | | 320 | Colon, both sexes | 1.07 (0.87-1.32) | |
| | | | | | | 136 | Rectum, both | 0.70 (0.51-0.95) | |
| **Hoffmeister *et al* (2007, Germany)** | Interview/ questionnaire, January, 2003- June, 2004 | Prevalent statin users (use at least 2 times a week for at least 1 year)/ Simvastatin, Lovastatin, Atorvastatin, Fluvastatin, Cerivastatin, Pravastatin | First diagnosis of cancer/ ICD 10 pos. C18-C20 | General population. Mean age cases: 68 Range age: ≥30-≥80 | 1154 | 540 | Colorectal, both sexes | 0.69 (0.45-1.06) | Yes/1.1-3,8, 14,15,17-19, 21-23,27, 29 |
| | | | | | | 324 | Colon, both sexes | 0.64 (0.39-1.04) | |
| | | | | | | 216 | Rectum, both sexes | 0.69 (0.37-1.28) | |

| Author (year, country) | Data source/ Study period | Exposure/ Active principles studied | Outcome/ Definition-confirmation | Definition of population | N | Cases | Site, sex | AOR (95% CI) | Matching/ Adjustment variables |
|---|---|---|---|---|---|---|---|---|---|
| **Vinogradova *et al* (2007, United Kingdom)** | Database (QRESEARCH database), January 1, 1995- July 31, 2005 | Prevalent statin users (at least 1 prescription during 13– 48 months before index date)/ Simvastatin, Atorvastatin, Fluvastatin, Cerivastatin, Pravastatin | First diagnosis of cancer/ Not defined | General population. Mean age cases: 72 (IQR 64-79) Range age: <55-≥85 | 30668 | 5686 | Colorectal, both sexes | 0.93 (0.83-1.04) | Yes/1,2,5,7, 8,10,14,17, 18,32,39,42-44 |
| **Boudreau *et al* (2008, United States)** | Database (Group Health Cooperative, Washington Surveillance, Epidemiology, and End Results cancer registry), January 1, 2000- December 31, 2003 | Prevalent statin users (at least 2 prescriptions during a 6 months period)/ Not defined/ Prevalent users | First diagnosis of cancer/ Not defined | General population. Mean age cases: 69.9 (SD± 12.3) Range age: ≥ 40 | 1330 | 665 | Colorectal, both sexes | 1.02 (0.65-1.59) | Yes/1,2,4,8, 11-18 |
| | | | | | | 512 | Colon, both sexes | 0.91 (0.55-1.50) | |
| | | | | | | 153 | Rectum, both sexes | 1.47 (0.50-4.29) | |
| **Yang *et al* (2008, United Kingdom)** | Database (GPRD), May, 1987- April, 2002 | Prevalent statin users (at least 5 years)/ Simvastatin, Lovastatin, Atorvastatin, Fluvastatin, Cerivastatin, Pravastatin | First diagnosis of cancer/ Diagnostic codes for CRC in the GPRD | General population. Mean age cases: 67.5 (SD± 8.9) | 48724 | 4432 | Colorectal, both sexes | 1.10 (0.50-2.20) | Yes/3,4,7, 14,15,17,18, 27,28 |
| **Hachem *et al* (2009, United States)** | Database (Department of Veterans Affairs), 1997-2002 | Prevalent statin users (at least 2 years before index date)/ Simvastatin | First diagnosis of cancer/ ICD-9 (codes 153.0/9, 154.0 /1/8, 230.3/4) | Population with diabetes mellitus. Mean age: 74 | 30400 | 6080 | Colorectal, both sexes | 0.91 (0.86-0.96) | Yes/1,2,14, 28,31,33-36 |
| **Shadman *et al* (2009, United States)** | Interview/ questionnaire, database (Wisconsin cancer reporting system), 1999-2001 | Prevalent statin users (at least 1 month within one year before cancer diagnosis)/ Simvastatin, Lovastatin, Fluvastatin, Pravastatin | Diagnosis of cancer/ Not defined | General female population. Range age: 50-74 | 2044 | 669 | Colorectal, women | 1.17 (0.74-1.85) | Yes/1,15,17, 18,22,27-29 |

| Author (year, country) | Data source/ Study period | Exposure/ Active principles studied | Outcome/ Definition-confirmation | Definition of population | N | Cases | Site, sex | AOR (95% CI) | Matching/ Adjustment variables |
|---|---|---|---|---|---|---|---|---|---|
| **Robertson *et al* (2010, Denmark)** | Database (National Health Service), January 1, 1991- December 31, 2008 | Prevalent statin users (at least 2 prescriptions)/ Simvastatin, Lovastatin, Atorvastatin, Fluvastatin, Pitavastatin, Cerivastatin, Pravastatin, Rosuvastatin | First diagnosis of cancer/ ICD-8: 153.00/99 154.00 /19; ICD-10: C18.0/9, C19.9, C20.9 | General population. Mean age: 71.20 | 109769 | 9979 | Colorectal, both sexes | 0.87 (0.80-0.96) | Yes/1-3,8, 14,19,31,37-39 |
| **Cheng *et al* (2011, Taiwan)** | Database (National Health Insurance), January, 1996- December, 2008 | Prevalent statin users (at least 1 prescription within one year before cancer diagnosis)/ Simvastatin, Lovastatin, Atorvastatin, Fluvastatin, Pravastatin, Rosuvastatin | First diagnosis of cancer/ ICD-9-CM: Code 153-154 | General population. Mean age cases: 68.34 (SD±10.40) Range age: ≥50 | 5780 | 1156 | Colorectal, both sexes | 1.09 (0.91-1.30) | Yes/1,2,5,8, 9,14,16,27, 31,33,35,40, 41 |
| **Samadder *et al* (2011, Israel)** | Interview/ questionnaire, Database (Clalit Health Services) 1998- 2004 | Prevalent statin users (use at least for 5 years). Reference category: less than 5 years or no use/ Simvastatin, Pravastatin | Diagnosis of cancer/ Standardized pathological review | Patients with IBD. Mean age cases: 69.8 (SD±11.8) | 3842 | 39 | Colorectal, both sexes | 0.10 (0.01, 1.31) | Yes/1-3,6, 17,20, 22,23 |
| | | | | Patients without IBD. Mean age cases: 69.8 (SD±11.8) | | 1882 | Colorectal, both sexes | 0.55 (0.43-0.70) | |
| **Vinogradova *et al* (2011, United Kingdom)** | Database (QResearch database), January 1, 1998- July 1, 2008 | Prevalent statin users (at least 2 prescriptions in 2 months)/ Simvastatin, Atorvastatin, Fluvastatin, Cerivastatin, Pravastatin, Rosuvastatin | First diagnosis of cancer/ READ codes (B13, B14) | General population. Mean age cases: 69 (IQR 60-77) Range age: ≥ 30 | 60373 | 11749 | Colorectal, both sexes | 1.07 (1.00-1.15) | Yes/1,2,5,7, 8,14,17,18, 26,37,39,42, 43,44 |
| **Broughton *et al* (2012, United Kingdom)** | Interview/ questionnaire, September 1, 2009- May 31, 2010 | Prevalent statin users/ Simvastatin, Atorvastatin, Pravastatin, Rosuvastatin | First diagnosis of cancer/ Histological confirmation | Population of Gastroenterology Department. Mean age cases: 70.3 (SD±10.2) Range age: ≥ 18 | 233 (62.40) | 101 | Colorectal, both sexes | 0.43 (0.25-0.80) | No/1,2,8,14, 19,36 |

| Author (year, country) | Data source/ Study period | Exposure/ Active principles studied | Outcome/ Definition-confirmation | Definition of population | N | Cases | Site, sex | AOR (95% CI) | Matching/ Adjustment variables |
|---|---|---|---|---|---|---|---|---|---|
| **Lakha *et al* (2012, United Kingdom)** | Interview/ questionnaire, Database (Health Informatics Centre), 1999-2009 | Prevalent statin users (1/2 prescriptions in 2 months pre-recruitment; 1/2 prescriptions in7 months pre-recruitment)/Not defined | First diagnosis of cancer/ Surgical units | General population. Statin use: ≥2 p at least 2 months pre-recruitment. Mean age cases: 60.0 (SD± 11.8). Range: 16–79 | 603 | 309* | Colorectal, both sexes | 0.42 (0.19-0.92) | Yes/1-3,14, 17,18,20,22, 33 |
| | | | | General population. Statin use: ≥2 p at least 7 months pre-recruitment. Mean age cases: 60.0 (SD± 11.8). Range: 16–79 | | 309* | Colorectal, both sexes | 0.49 (0.22-1.08) | |
| **Deshpande *et al* (2013, United States)** | Database (HealthCore Integrated Research Database), January 1, 2001- January 30, 2011 | Prevalent statin users (at least one prescription)/ Simvastatin, Lovastatin, Atorvastatin, Fluvastatin, Cerivastatin, Pravastatin, Rosuvastatin | First diagnosis of cancer/ ICD-9 (codes 153.xx, 230.3 or 230.4) | General population. Mean age cases: 60.7 (SD± 13.2) Range age: ≥18 | 73472 | 36736 | Colon, both sexes | 0.96 (0.89-1.05) | Yes/1,2,4,5, 27,33 |
| **Björkhem-Bergman *et al* (2014, Sweden)** | Database (Swedish Cancer Register, Swedish Prescribed Drug Register), July 1, 2006- December 31, 2010 | Prevalent statin users (at least 9 months during the year before of index date)/ Simvastatin, Atorvastatin, Fluvastatin, Pravastatin, Rosuvastatin | First diagnosis of cancer/ ICD-0/3 code C18 | General population. Range age: 40-≥90 | 126858 | 21143 | Colon, both sexes | 1.04 (1.00-1.08) | Yes/1,2,5,14 ,24,29,33,45 |
| **Sehdev *et al* (2014, United States)** | Database (MarketScan), 2004-2010 | Prevalent statin users (statin use measured during a 12-month period prior to diagnosis)/ Simvastatin, Lovastatin, Atorvastatin, Pravastatin, Rosuvastatin | First diagnosis of cancer/ ICD9-CM codes (153.0-9, 154.0/1/8) | General population. Mean age cases: 54.44 (SD± 7.5) | 357702 | 32616 | Colorectal, both sexes | 0.74 (0.72-0.77) | Yes/1.1-3,5, 7-10,14,18, 33,36,42,46 |

| Author (year, country) | Data source/ Study period | Exposure/ Active principles studied | Outcome/ Definition-confirmation | Definition of population | N | Cases | Site, sex | AOR (95% CI) | Matching/ Adjustment variables |
|---|---|---|---|---|---|---|---|---|---|
| **Mamtani *et al* (2016, United Kingdom)** | Database (THIN), 1995-2013 | Prevalent statin users / Not defined | First diagnosis of cancer/ READ codes (B13x, B803x, B804x, B14x) | General population. Short-term (< 1 yr). Mean age cases: 72.3 (IQR 63.8-79.6) | 108701 | 904 | Colorectal, both sexes | 0.92 (0.85-0.99) | Yes/1.1-4, 8,14-19,27, 28 |
| | | | | General population. Long-term (> 1 yr). Mean age cases: 72.3 (IQR 63.8-79.6) | | 4936 | Colorectal, both sexes | 0.95 (0.91-0.99) | |
| **Horibe *et al* (2017, Japan)** | Interview/ questionnaire, August 2012, June 2014 | Prevalent statin users / Simvastatin, Atorvastatin, Fluvastatin, Pitavastatin, Pravastatin, Rosuvastatin | Diagnosis of cancer/ Colonoscopy | Population with diabetes mellitus who underwent total colonoscopy. Mean age: 71.6 (SD±10.1) | 168 | 70 | Colorectal, both sexes | 4.59 (1.69-13.43) | No/1,2,13, 14,16-19,36, 48,59,60 |
| **Renman *et al* (2017, Sweden)** | Database (electronic health records), January 1, 2012-December 31, 2015 | Prevalent statin users (at least 2 prescriptions, one of them more than 1-year prior cancer diagnosis)/ Simvastatin, Atorvastatin | First diagnosis of cancer/ ICD-10 (codes C18-C20) | General population. Mean age cases: 70.4 (SD± 12.7) | 325 | 107 | Colorectal, both sexes | 1.12 (0.64-1.96) | Yes/1,8 |
| **Ibáñez-Sanz *et al* (2019, Spain)** | Database (SIDIAP), January 1, 2010-December 31,2015 | Prevalent statin users (at least one prescription)/ Simvastatin, Lovastatin, Atorvastatin, Fluvastatin, Pravastatin, Rosuvastatin | First diagnosis of cancer/ ICD-10 (C18, C19, C20) | General population. Mean age users: 74.0 Range age: 18-90 | 154928 | 25811 | Colorectal, both sexes | 0.98 (0.95-1.01) | Yes/1.1-3,5, 10,14,17-19,32 |
| | | | | | | 21242 | Colon, both sexes | 1.00 (0.97-1.03) | |
| | | | | | | 4569 | Rectum, both sexes | 0.87 (0.81-0.92) | |
| **Dickerman *et al* (2020, United Kingdom)** | Database (Clinical Practice Research Database), January, 1999 – February, 2016 | New statin users (no statin prescriptions within the past year)/ Not defined | First diagnosis of cancer/ Read codes in primary care and as ICD-10 codes in hospitals | General population. Mean age cases: 68.5 (SD± 10.7) | 3599596 | 3596 | Colorectal, both sexes | 1.00 (0.86-1.16) | No/1,2,7,14, 15,17,18,21, 42,43,47 |

1: year of birth, 1.1: 5-year age-group, 2: sex, 3: geographic region, 4: duration of follow up, 5: index date, 6: ethnic group/race, 7: practice, 8: diabetes mellitus, 9: hospitalization, 10: comorbidity, 11: diuretics, 12: ACE inhibitors, 13: calcium-channel blockers, 14: NSAIDs, 15: hormone therapy, 16: other lipid-lowering therapies, 17: smoking, 18: BMI, 19: alcohol use, 20: exercise, 21: hypercholesterolemia/hyperlipidemia, 22: family history of colorectal cancer, 23: diet (vegetable consumption/red meat), 24: glucocorticosteroids, 25: immunomodulators, 26: 5-ASAs, 27: colorectal cancer endoscopic screening, 28: calendar year, 29: educational level, 30: vitamins and calcium supplements, 31: cholecystectomy, 32: socioeconomic status, 33: inflammatory bowel disease, 34: nephropathy, 35: liver disease, 36: antidiabetics agents, 37: Myocardial infarction, 38: Atherosclerosis, 39: Stroke, 40: polyps, 41: fecal occult blood, 42: coronary heart disease, 43: hypertension, 44: rheumatoid arthritis, 45: chemotherapy, 46: polycystic ovary disease, 47: cerebrovascular disease, 48: other

cardiovascular drugs, 49: thyroid disease. 50: mental illness, 51: lung disease 52: gastrointestinal disease 53: prostate disease 54: immunomodulator, 55: anti-TNF biologic exposure, 56: glucose, 57: previous benign neoplasm, 58: year of cohort entry, 59: HbA1c values, 60: fecal immunochemical test

*Number of exposed cases

## b) Cohort studies

| Author (year, country) | Data source/ Study period | Exposure/ Active principles studied | Outcome/ Definition-confirmation | Definition of population | N | Cases | Site, sex | RR/HR/IRR/OR (95% CI) | Matching/ Adjustment variables |
|---|---|---|---|---|---|---|---|---|---|
| **Friis *et al* (2005, Denmark)** | Database (Central Population Register, Pharmacoepide-miological Prescription Database), January 1, 1989- December 31, 2002 | Prevalent statin users. Reference category: non-statin lipid-lowering drugs users (at least 2 prescriptions). Subjects are allowed to change exposure variables/ Simvastatin, Lovastatin, Atorvastatin, Fluvastatin, Cerivastatin, Pravastatin | First diagnosis of cancer/ Not defined | General population. Mean age statin users: 60.7 Range age: 30-80 | 334754 | 3006 | Colorectal, both sexes | RR: 0.85 (0.65-1.11) | No/1,2,12-15,28,48 |
| **Jacobs *et al* (2006, United States)** | Interview/ questionnaire, 1997-2001 | Prevalent statin users/ Simvastatin, Lovastatin, Fluvastatin, Pravastatin | First diagnosis of cancer/ Not defined | General population. Range age: ≤50 ≥80 | 132136 | 815 | Colorectal, both sexes | RR: 1.03 (0.85-1.26) | No/1,2,6,14, 15,18,20,21, 23,27,29,37 |
| **Setoguchi *et al* (2007, United States)** | Database (Pharmaceutical Assistance Contract for the Elderly, Medicare and Pennsylvania State Cancer Registry, Medicare Current Beneficiary Survey), January 1,1994- May 31, 2003 | New statin users (no statin prescriptions within the past year) (at least 3 prescriptions in 6 months). Non-exposed: Users of glaucoma drugs/ Simvastatin, Lovastatin, Atorvastatin, Fluvastatin, Cerivastatin, Pravastatin | First diagnosis of cancer/ Not defined | General population. Mean age statin users: 76.4 (SD± 6.0) Range age: ≥65 | 31723 | 249 | Colorectal, both sexes | HR: 0.96 (0.70-1.31) | No/1,2,6-10, 14,15,17,18, 27,33,40,44 |
| **Farwell *et al* (2008, United States)** | Database (VA VISN-1), January 1, 1997-December 31, 2005 | Prevalent users of statin and antihypertensive drug use (at least 2 prescriptions within 1 year). Non-exposed: Nonusers of statins, but users of antihypertensive drugs/ Simvastatin, Lovastatin, Atorvastatin, Fluvastatin, Pravastatin | First diagnosis of cancer/ ICD-9-CM (codes 140.XX – 208.XX) | General population. Mean age statin users: 66.6 (SD± 10) Range age: ≥18 | 62842 | 687 | Colorectal, both sexes | HR: 0.65 (0.55-0.78) | No/1,8,14,17 -19,21,27, 34,42,43,49-53 |

| Author (year, country) | Data source/ Study period | Exposure/ Active principles studied | Outcome/ Definition-confirmation | Definition of population | N | Cases | Site, sex | RR/HR/IRR/OR (95% CI) | Matching/ Adjustment variables |
|---|---|---|---|---|---|---|---|---|---|
| **Friedman *et al* (2008, United States)** | Database (Kaiser Permanente Medical Care Program, Pharmacy Information Management System), August, 1994 -December, 2003 | New statin users (all persons were considered to be non-users of statins until one was dispensed to them) (2 years before diagnosis)/ Simvastatin, Lovastatin, Atorvastatin, Fluvastatin, Cerivastatin, Pravastatin, Rosuvastatin | First diagnosis of cancer/ Not defined | General male population. Range age: 20-79 | 2097474 | 292* | Colon, men | RR: 0.88 (0.78-1.00) | No/28 |
| | | | | | | 129* | Rectum, men | RR: 0.93 (0.77-1.12) | |
| | | | | General female population. Range age: 20-79 | 2145593 | 243* | Colon, women | RR: 0.97 (0.85-1.11) | |
| | | | | | | 69* | Rectum, women | RR: 0.97 (0.76-1.25) | |
| **Flick *et al* (2009, United States)** | Interview/ questionnaire, 1991-2005 | Prevalent statin users (at least 1 prescription in 3 months)/ Simvastatin, Lovastatin, Atorvastatin, Fluvastatin, Cerivastatin, Pravastatin, Rosuvastatin | First diagnosis of cancer/ Not defined | General male population. Mean age: 58<br>Range age: 45–69 | 69115 | 171 | Colorectal, men | HR: 0.89 (0.61-1.30) | No/1,14,17-23,27,30,40, 42 |
| | | | | | | 123 | Colon, men | HR: 0.90 (0.58-1.40) | |
| | | | | | | 48 | Rectum, men | HR: 0.86 (0.41-1.78) | |
| **Singh *et al* (2009, United States)** | Database (Manitoba Cancer Registry and Drug Program Information, MHHL Population Registry and Hospital Discharge Abstract and Medical Claims database), April 1,1995- December 31, 2005 | Prevalent statin users (at least 2 prescriptions in 3 months)/ Simvastatin, Lovastatin, Atorvastatin, Fluvastatin, Cerivastatin, Pravastatin, Rosuvastatin | Diagnosis of cancer/ Not defined | General population. Mean age: 62<br>Range age: ≥40 | 423558 | 6637 | Colorectal, both sexes | IRR: 1.13 (1.02-1.25) | No/1,2,8,10, 14,15,27,32, 33,42 |

| Author (year, country) | Data source/ Study period | Exposure/ Active principles studied | Outcome/ Definition-confirmation | Definition of population | N | Cases | Site, sex | RR/HR/IRR/OR (95% CI) | Matching/ Adjustment variables |
|---|---|---|---|---|---|---|---|---|---|
| **Haukka *et al* (2010, Finland)** | Database (Social Insurance Institution, Finnish Cancer Registry), January 1, 1996- December 31, 2005 | Prevalent statin users (at least one prescription)/ Simvastatin, Lovastatin, Atorvastatin, Fluvastatin, Cerivastatin, Pravastatin, Rosuvastatin | First diagnosis of cancer/ ICD-7 (153, 154) | General population. Mean age: 60 (IQR 52.6- 67.9) | 472481 | 2950 | Colon, both sexes | RR: 0.99 (0.92-1.06) | Yes/1-3,4 |
| | | | | | | 2066 | Rectum, both sexes | RR: 1.07 (0.98-1.17) | |
| **Jacobs *et al* (2011, United States)** | Interview/ questionnaire, database (state cancer registries) 1997-2007 | Prevalent statin users/ Simvastatin, Lovastatin, Fluvastatin, Pravastatin | First diagnosis of cancer/ Not defined | General population. Statin use: <5 years Range age: <60-≥80 | 133255 | 270* | Colorectal, both sexes | RR: 0.96 (0.82-1.12) | No/1,2.6,8, 14,15,17,18, 20,21,27,29, 42,43 |
| | | | | General population. Statin use: ≥5 years Range age: <60-≥80 | | 259* | Colorectal, both sexes | RR: 0.93 (0.80-1.07) | |
| **Lee *et al* (2011, United States)** | Interview/ questionnaire, database (National Death Index), 1990-2006 | Prevalent statin users/ Not defined | First diagnosis of cancer/ Standardized pathological review | General population. Mean age men: 67.4-68.7 Mean age women: 67.7-69.9 | 131922 | 1680 | Colorectal, both sexes | RR: 0.97 (0.84-1.12) | No/1,14,17-19, 22,23,27, 28,29 |
| | | | | | | 1282 | Colon, both sexes | RR: 1.10 (0.94-1.29) | |
| | | | | | | 398 | Rectum, both sexes | RR: 0.59 (0.41-0.84) | |
| **Clancy *et al* (2013, Italy)** | Database (Emilia-Romagna Region (RER) health care database), 2003-2010 | New statin users (no prescriptions within the past year) (at least 3 prescriptions in 6 months). Non-exposed: new users of glaucoma drugs (no prescriptions within the past year) / Simvastatin, Lovastatin, Atorvastatin, Fluvastatin, Pitavastatin, Cerivastatin, Pravastatin, Rosuvastatin | First diagnosis of cancer/ ICD-9 (codes 153.xx, 230.3 or 230.4) | General population. Mean age statin users: 64.6 (SD±11.4) | 266109 | 2420 | Colorectal, both sexes | HR: 0.84 (0.76-0.93) | Yes (Propensity score)/1,2,9, 10,14,27,33, 48 |

| Author (year, country) | Data source/ Study period | Exposure/ Active principles studied | Outcome/ Definition-confirmation | Definition of population | N | Cases | Site, sex | RR/HR/IRR/OR (95% CI) | Matching/ Adjustment variables |
|---|---|---|---|---|---|---|---|---|---|
| **Leung *et al* (2013, Taiwan)** | Database (National Health Insurance Research Database), January 1, 2000- December 31, 2008 | Prevalent statin users (continuously use for at least 6 months)/ Simvastatin, Atorvastatin, Fluvastatin, Pravastatin, Rosuvastatin | First diagnosis of cancer/ ICD-9-CM (153.xx, 154.xx) | General population. Mean age: 58.31 (SD±12.12) | 34205 | 458 | Colon, both sexes | HR: 1.11 (0.77-1.60) | Yes/1,2,5,8, 10,14-16 |
| | | | | | | 196 | Rectum, both sexes | HR: 0.54 (0.27-1.12) | |
| **Ananthakrishnan *et al* (2016, United States)** | Database (electronic health records), 1998-2010 | Prevalent statin users (at least one prescription)/ Simvastatin, Lovastatin, Atorvastatin, Fluvastatin, Pitavastatin, Pravastatin, Rosuvastatin | Diagnosis of cancer/ ICD-9-CM (153.x – 154.x) | Patients with IBD. Mean age statin users: 67 (IQR 58-76) | 11001 | 317 | Colorectal, both sexes | OR: 0.42 (0.28-0.62) | No/1,2,4,6, 17,27,33,54, 55 |
| **Liu *et al* (2016, Taiwan)** | Database (National Health Insurance Research Database), January 1, 2001- December 31, 2012 | Prevalent statin users/ Simvastatin, Lovastatin, Atorvastatin, Fluvastatin, Pravastatin, Rosuvastatin | First diagnosis of cancer/ ICD-9-CM (codes 153.0–153.9) | Patients with Chronic Obstructive Pulmonary Disease. Mean age: 62.92 (SD±13.18) | 43802 | 946 | Colon, both sexes | HR: 0.52 (0.44-0.62) | Yes (Propensity score)/1,2,8, 10,21,32,43 |
| | | | | | | 591 | Colon, men | HR: 0.49 (0.39-0.63) | |
| | | | | | | 355 | Colon, women | HR: 0.55 (0.42-0.72) | |
| **Lee *et al* (2019, Korea)** | Database (National Health Insurance Service-Health Screening) 2002-2015 | Prevalent statin users (statin use during 2002-2003). Non-exposed: nonusers of statins despite having hypercholesterolemia/ Simvastatin, Lovastatin, Atorvastatin, Fluvastatin, Cerivastatin, Pitavastatin, Pravastatin, Rosuvastatin | First diagnosis of cancer/ ICD-10 (code C18.0-C20.0) | Hypercholesterolemia (cholesterol ≥250 mg/dL). Low users (statin usage). Mean age: 53.4 (SD±0.1) | 31149 | 617† | Colorectal, both sexes | HR: 1.10 (0.86-1.40) | No/1,8,17-21, 32,43,56 |
| | | | | Hypercholesterolemia (cholesterol ≥250 mg/dL). High users (statin usage). Mean age: 54.5 (SD±0.1) | | 617† | Colorectal, both sexes | HR: 0.60 (0.45-0.80) | |

| Author (year, study location) | Data source/ Study period | Exposure/ Active principles studied | Outcome/ Definition-confirmation | Definition of population | N | Cases | Site, sex | RR/HR/IRR/OR (95% CI) | Matching/ Adjustment variables |
|---|---|---|---|---|---|---|---|---|---|
| **Erkinantti *et al* (2021, Finland)** | Database (FinDM-Diabetes in Finland database)/ January 1, 1996- December 31, 2011 | Prevalent statin users/Not defined | First diagnosis of cancer/ ICD-10 (C18) ICD-O-3 (M-8140/3) | Patients with type 2 Diabetes. Range age: 40-107 years | 306317 | 1349 | Colon, both sexes | HR: 0.94 (0.84-1.05) | No/1,2,8 |
| **Han *et al* (2021, Korea)** | Database (National Health Insurance Service of South Korea)/2009-2011 | Prevalent statin users/Not defined | First diagnosis of cancer/ ICD-10 (C18-C20) | General population. Mean age: 70.75 (SD± 2.26) | 131266 | 1552 | Colorectal, both sexes | HR: 0.80 (0.67-0.95) | No/1-3,8,17-19,22,61 |
| **Yeh *et al* (2021, Taiwan)** | Database (Taiwan's National Health Insurance)/ January 1, 2000- December 31, 2013 | Prevalent and new users (use less than 3 months)/ Not defined | First diagnosis of cancer/ ICD-9-CM (codes 153-154) | Patients with Interstitial lung disease and pulmonary fibrosis. Mean age: 55.3 (SD±16.7) | 20072 | 380 | Colorectal, both sexes | HR: 0.73 (0.59-0.89) | Yes (propensity score)/1,2,8, 10,18,21,24, 34,39,43,50 |
| **Zhang *et al* (2022, United States)** | Interview/ questionnaire, database (National Death Index)/1990-2016 | Prevalent statin users/Not defined | First diagnosis of cancer/ Medical and pathology reports, state cancer registries | General population. Mean age women: 67.0 (SD±9.0)- 78.6 (SD±6.4) (duration of statin use) Mean age men: 65.0 (SD±10.8)- 76.7 (SD±7.6) (duration of statin use) | 148291 | 2924 | Colorectal, both sexes | HR: 1.05 (0.95-1.16) | No/1,2,6,8,14,17-22,23, 30 |

1: year of birth, 1.1: 5-year age-group, 2: sex, 3: geographic region, 4: duration of follow up, 5: index date, 6: ethnic group/race, 7: practice, 8: diabetes mellitus, 9: hospitalization, 10: comorbidity, 11: diuretics, 12: ACE inhibitors, 13: calcium-channel blockers, 14: NSAID, 15: hormone therapy, 16: other lipid-lowering therapies, 17: smoking, 18: BMI, 19: alcohol use, 20: exercise, 21: hypercholesterolemia/hyperlipidemia, 22: family history of colorectal cancer, 23: diet (vegetable consumption/red meat), 24: glucocorticosteroids, 25: immunomodulators, 26: 5-ASAs, 27: colorectal cancer endoscopic screening, 28: calendar year, 29: educational level, 30: vitamins and calcium supplements, 31: cholecystectomy, 32: socioeconomic status, 33: inflammatory bowel disease, 34: nephropathy, 35: liver disease, 36: antidiabetics agents, 37: Myocardial infarction, 38: Atherosclerosis, 39: Stroke, 40: polyps, 41: fecal occult blood, 42: coronary heart disease, 43: hypertension, 44: rheumatoid arthritis, 45: chemotherapy, 46: polycystic ovary disease, 47: cerebrovascular disease, 48: other cardiovascular drugs, 49: thyroid disease. 50: mental illness, 51: lung disease 52: gastrointestinal disease 53: prostate disease 54: immunomodulator, 55: anti-TNF biologic exposure, 56: glucose, 57: previous benign neoplasm, 58: year of cohort entry, 59: HbA1c values, 60: fecal immunochemical test, 61: insurance

*Number of exposed cases

†Total number of cases (not disaggregated)

## Table S2. Reported subgroup analyses from studies included in review

### a) Case-control studies

| Author (year, country) | Sex categories | | Age categories | Duration categories | | | Intensity categories | Lipophilicity/ Active principle categories | |
|---|---|---|---|---|---|---|---|---|---|
| **Blais *et al* (2000, Canada)** | Not reported | | Not reported | Not reported | | | Not reported | Not reported | |
| **Graaf *et al* (2004, Netherlands)** | Not reported | | Not reported | Not reported | | | Not reported | Not reported | |
| **Kaye *et al* (2004, United Kingdom)** | Not reported | | Not reported | Colon | <2 years | 0.80 (0.40-1.90) | Not reported | Not reported | |
| | | | | | 2-5 years | 0.90 (0.40-1.80) | | | |
| | | | | | ≥5 years | 3.50 (1.10-10.90) | | | |
| | | | | Rectum | <2 years | 1.20 (0.50-3.00) | | | |
| | | | | | 2-5 years | 1.40 (0.60-3.20) | | | |
| | | | | | ≥5 years | 4.20 (1.10-16.60) | | | |
| **Khurana *et al* (2004, United States)** | Not reported | | Not reported | Not reported | | | Not reported | Not reported | |
| **Poynter *et al* (2005, Israel)** | Not reported | | Not reported | ≥5 years | | 0.55 (0.40-0.74) | Not reported | Simvastatin | 0.49 (0.36-0.67)* |
| | | | | | | | | Pravastatin | 0.44 (0.31-0.63)* |
| **Rubin *et al* (2005, Denmark)** | Not reported | | Not reported | Not reported | | | Not reported | Not reported | |
| **Coogan *et al* (2007, United States)a** | Not reported | | Not reported | ≤1 year | | 0.70 (0.30-1.50) | Not reported | Not reported | |
| | | | | >1-<5 years | | 0.70 (0.40-1.30) | | | |
| | | | | ≥5 years | | 1.10 (0.60-2.20) | | | |
| **Coogan *et al* (2007, United States)b** | Both sexes | 0.92 (0.78-1.09) | Not reported | Regular use | | 0.92 (0.78-1.09) | Not reported | Simvastatin | 0.81 (0.59-1.12) |
| | | | | | | | | Lovastatin | 1.73 (1.03-2.88) |
| | | | | | | | | Atorvastatin | 0.93 (0.76-1.14) |

| | | | | | | | | | |
|---|---|---|---|---|---|---|---|---|---|
| | | | | | | | | Pravastatin | 0.94 (0.60-1.46) |
| | | | | | ≤1 year | 0.57 (0.27-1.24) | | | |
| | | | | | >1-4 years | 0.94 (0.77-1.15) | | | |
| | | | | | 5-9 years | 0.95 (0.71-1.27) | | | |
| | | | | | ≥ 10 years | 0.86 (0.51-1.45) | | | |
| | Women | 1.02 (0.78-1.32) | | | | | | | |
| | Men | 0.86 (0.68-1.08) | | | | | | | |
| **Hoffmeister *et al* (2007, Germany)** | Not reported | | Not reported | | 1-4 years | 0.58 (0.33-1.04) | Not reported | Simvastatin | 0.50 (0.24-1.02) |
| | | | | | | | | Atorvastatin | 0.84 (0.46-1.53) |
| | | | | | ≥5 years | 0.71 (0.39-1.28) | | Pravastatin | 0.61 (0.21-1.81) |
| **Vinogradova *et al* (2007, United Kingdom)** | Not reported | | Not reported | | < 1 year | 0.84 (0.71-1.00) | Not reported | Simvastatin | 0.83 (0.72-0.96) |
| | | | | | | | | Atorvastatin | 1.11 (0.95-1.30) |
| | | | | | 1-2 years | 0.99 (0.80-1.22) | | Fluvastatin | 1.21 (0.85-1.74) |
| | | | | | | | | Cerivastatin | 1.34 (0.97-1.86) |
| | | | | | > 2 years | 0.99 (0.84-1.16) | | Pravastatin | 0.84 (0.55-1.28) |
| **Boudreau *et al* (2008, United States)** | Not reported | | Not reported | | < 2 years | 0.80 (0.40-1.59) | Not reported | Not reported | |
| | | | | | ≥2 years | 1.22 (0.70-2.12) | | | |
| **Yang *et al* (2008, United Kingdom)** | Not reported | | Not reported | | ≥5 years | 1.10 (0.50-2.20) | Not reported | Not reported | |
| | | | | | ≥10 years | 1.30 (0.60-2.70) | | | |
| **Hachem *et al* (2009, United States)** | Not reported | | ≥65 years | 0.90 (0.85-0.96) | < 0.5 year | 0.86 (0.77-0.95) | Not reported | Simvastatin | 0.91 (0.86-0.97) |
| | | | | | 0.5-1 year | 0.98 (0.89-1.09) | | | |
| | | | | | >1-1.5 year | 0.91 (0.82-1.02) | | | |
| | | | | | 1.5-2 years | 0.93 (0.84-1.04) | | | |
| | | | | | > 2 years | 0.87 (0.80-0.95) | | | |
| **Shadman *et al* (2009, United States)** | Women | 1.17 (0.74 -1.85) | Not reported | | < 3 years | 1.07 (0.56-2.03) | Not reported | Lipophilicity | 1.20 (0.72-2.00) |
| | | | | | ≥ 3 years | 1.27 (0.68-2.38) | | Hydrophilicity | 1.10 (0.44-2.77) |
| **Robertson *et al* (2010, Denmark)** | Not reported | | Not reported | | 0-3 years | 0.84 (0.75-0.95) | Not reported | Simvastatin | 0.88 (0.79-0.98) |
| | | | | | | | | Lovastatin | 0.78 (0.45-1.35) |
| | | | | | 3-5 years | 0.88 (0.74-1.04) | | Atorvastatin | 0.73 (0.52-1.01) |
| | | | | | > 5 years | 0.95 (0.80-1.12) | | Pravastatin | 0.85 (0.61-1.20) |
| **Cheng *et al* (2011, Taiwan)** | Not reported | | Not reported | | Not reported | | Not reported | Not reported | |

| Author (year, country) | Sex categories | Age categories | | Duration categories | | Intensity categories | Lipophilicity/ Active principle categories | |
|---|---|---|---|---|---|---|---|---|
| **Samadder *et al* (2011, Israel)** | Not reported | Not reported | | Not reported | | Not reported | Simvastatin, IBD | 0.05 (0.004-0.54) |
| | | | | | | | Pravastatin, IBD | 0.11 (0.004-3.00) |
| | | | | | | | Simvastatin, Non-IBD | 0.36 (0.30-0.42) |
| | | | | | | | Pravastatin, Non- IBD | 0.41 (0.31-0.52) |
| **Vinogradova *et al* (2011, United Kingdom)** | Not reported | Not reported | | <1 year | 1.05 (0.95-1.17) | Not reported | Simvastatin | 0.96 (0.88-1.04) |
| | | | | >1-2 years | 1.04 (0.92-1.17) | | | |
| | | | | >2-4 years | 1.02 (0.92-1.14) | | Atorvastatin | 1.17 (1.07-1.28) |
| | | | | >4 years | 1.23 (1.10-1.38) | | Pravastatin | 1.09 (0.93-1.28) |
| **Broughton *et al* (2012, United Kingdom)** | Not reported | Not reported | | <2 years | 0.66 (0.21-1.69) | Not reported | Simvastatin | 0.43 (0.10-0.73) |
| | | | | 2-5 years | 0.38 (0.14-1.01) | | | |
| | | | | >5 years | 0.18 (0.06-0.55) | | | |
| **Lakha *et al* (2012, United Kingdom)** | Not reported | Not reported | | Not reported | | Not reported | Not reported | |
| **Deshpande *et al* (2013, United States)** | Not reported | Not reported | | <0.5 years | 0.84 (0.75-1.24) | Not reported | Simvastatin | 0.94 (0.89-0.99) |
| | | | | | | | Fluvastatin | 0.84 (0.67-1.05) |
| | | | | <1 year | 0.86 (0.83-0.89) | | Cerivastatin | 1.05 (0.75-1.48) |
| | | | | | | | Pravastatin | 0.91 (0.84-0.99) |
| | | | | | | | Rosuvastatin | 0.97 (0.87-1.08) |
| **Björkhem-Bergman *et al* (2014, Sweden)** | Not reported | Not reported | | Not reported | | Not reported | Not reported | |
| **Sehdev *et al* (2014, United States)** | Not reported | ≤ 55 years | 0.67 (0.63-0.71) | 91-217 days | 0.77 (0.62-0.95) | Not reported | Simvastatin | 0.79 (0.75-0.83) |
| | | | | 218-303 days | 0.93 (0.67-1.02) | | | |
| | | > 55 years | 0.79 (0.76-0.82) | 304-365 days | 0.80 (0.65-0.99) | | | |

| Author (year, country) | Sex categories | | Age categories | | Duration categories | | Intensity categories | | Lipophilicity/ Active principle categories | |
|---|---|---|---|---|---|---|---|---|---|---|
| **Mamtani *et al* (2016, United Kingdom)** | Not reported | | Not reported | | < 1 year | 0.92 (0.85-0.99) | Not reported | | Not reported | |
| | | | | | > 1 year | 0.95 (0.91-0.99) | | | | |
| **Horibe *et al* (2017, Japan)** | Not reported | | Not reported | | Not reported | | Not reported | | Not reported | |
| **Renman *et al* (2017, Sweden)** | Not reported | | Not reported | | Not reported | | Not reported | | Not reported | |
| **Ibáñez-Sanz *et al* (2019, Spain)** | Women | 1.01 (0.97-1.05) | 18-55 years | 1.07 (0.95-1.20) | < 5 years | 1.11 (1.04-1.08) | Low | 0.98 (0.94-1.01) | Lipophilicity | 0.98 (0.95-1.01) |
| | | | | | | | | | Hydrophilicity | 0.96 (0.87-1.05) |
| | | | 55-65 years | 0.98 (0.91-1.04) | | | | | Simvastatin | 0.97 (0.94-0.99) |
| | | | | | | | | | Lovastatin | 1.04 (0.96-1.13) |
| | | | 65-75 years | 1.00 (0.95-1.06) | | | | | Atorvastatin | 0.99 (0.96-1.03) |
| | Men | 0.95 (0.91-0.98) | | | > 5 years | 0.96 (0.93-1.00) | High | 1.01 (0.96-1.07) | Fluvastatin | 0.98 (0.90-1.06) |
| | | | 75-85 years | 0.96 (0.92-1.01) | | | | | Pitavastatin | 1.03 (0.76-1.34) |
| | | | | | | | | | Pravastatin | 0.98 (0.93-1.04) |
| | | | 85-95 years | 0.96 (0.88-1.05) | | | | | Rosuvastatin | 0.92 (0.82-1.03) |
| **Dickerman *et al* (2020, United Kingdom)** | Not reported | | Not reported | | Not reported | | Not reported | | Not reported | |

*Non-adjusted odds ratios
Coogan et al (2007, United States)a: Statin Use and the Risk of 10 Cancers. DOI: 10.1097/01.ede.0000254694.03027.a1
Coogan et al (2007, United States)b: Statin Use and Risk of Colorectal Cancer. DOI: 10.1093/jnci/djk003

## b) Cohort studies

| Author (year, country) | Sex categories | | Age categories | Duration categories | | Intensity categories | Lipophilicity/ Active principle categories | |
|---|---|---|---|---|---|---|---|---|
| **Friis *et al* (2005, Denmark)** | Not reported | | Not reported | Not reported | | Not reported | Not reported | |
| **Jacobs *et al* (2006, United States)** | Not reported | | Not reported | <5 years | 1.00 (0.80-1.25) | Not reported | Not reported | |
| | | | | ≥5 years | 1.09 (0.83-1.43) | | | |
| **Setoguchi *et al* (2007, United States)** | Not reported | | Not reported | < 3 years | 0.93 (0.57-1.51) | Not reported | Not reported | |
| | | | | ≥ 3 years | 0.97 (0.65-1.46) | | | |
| **Farwell *et al* (2008, United States)** | Not reported | | Not reported | Not reported | | Not reported | Not reported | |
| **Friedman *et al* (2008, United States)** | Colon, women | 0.97 (0.85-1.11) | Not reported | > 5 years | 1.02 (0.75-1.38) | Not reported | Not reported | |
| | Rectum, women | 0.97 (0.76-1.25) | | > 5 years | 1.15 (0.66-2.01) | | | |
| | Colon, men | 0.88 (0.78-1.00) | | > 5 years | 1.00 (0.78-1.30) | | | |
| | Rectum, men | 0.93 (0.77-1.12) | | > 5 years | 1.09 (0.74-1.61) | | | |
| **Flick *et al* (2009, United States)** | Men | 0.89 (0.61-1.30) | Not reported | 101 days-<5 years | 0.91 (0.61-1.34) | Not reported | Not reported | |
| | | | | > 5 years | 0.83 (0.43-1.63) | | | |
| **Singh *et al* (2009, United States)** | Not reported | | Not reported | ≥5 years | 0.89 (0.70-1.13) | Not reported | Not reported | |
| **Haukka *et al* (2010, Finland)** | Not reported | | Not reported | Colon, 1 year | 1.01 (0.98-1.04) | Not reported | Simvastatin | 0.99 (0.96-1.01) |
| | | | | | | | Lovastatin | 0.97 (0.94-1.00) |
| | | | | | | | Atorvastatin | 1.03 (1.00-1.06) |
| | | | | | | | Fluvastatin | 0.98 (0.94-1.02) |
| | | | | | | | Pravastatin | 1.02 (0.97-1.08) |
| | | | | Rectum, 1 year | 0.96 (0.92-0.99) | | Simvastatin | 1.00 (0.97-1.03) |
| | | | | | | | Lovastatin | 0.94 (0.90-0.98) |
| | | | | | | | Atorvastatin | 1.02 (0.98-1.06) |
| | | | | | | | Fluvastatin | 0.98 (0.93-1.03) |

| Author (year, country) | Sex categories | | Age categories | | Duration categories | | Intensity categories | Lipophilicity/ Active principle categories | |
|---|---|---|---|---|---|---|---|---|---|
| | | | | | | | | Pravastatin | 0.91 (0.84-0.99) |
| **Jacobs *et al* (2011, United States)** | Not reported | | Not reported | | < 5 years | 0.93 (0.80-1.07) | Not reported | Not reported | |
| | | | | | ≥ 5 years | 0.96 (0.82-1.12) | | | |
| **Lee *et al* (2011, United States)** | Not reported | | Not reported | | 0-2 years | 0.99 (0.82-1.20) | Not reported | Not reported | |
| | | | | | 3-5 years | 1.01 (0.79-1.28) | | | |
| | | | | | ≥6 years | 0.97 (0.75-1.25) | | | |
| **Clancy *et al* (2013, Italy)** | Both sexes | 0.84 (0.76-0.93) | Not reported | | Not reported | | Not reported | Lipophilicity | 0.84 (0.76-0.94) |
| | | | | | | | | Hydrophilicity | 0.85 (0.75-0.96) |
| | Women | 0.95 (0.82-1.10) | | | | | | Lipophilicity | 0.93 (0.79-1.08) |
| | | | | | | | | Hydrophilicity | 0.99 (0.82-1.19) |
| | Men | 0.77 (0.67-0.88) | | | | | | Lipophilicity | 0.79 (0.69-0.90) |
| | | | | | | | | Hydrophilicity | 0.75 (0.63-0.89) |
| **Leung *et al* (2013, Taiwan)** | Not reported | | Not reported | | Not reported | | Not reported | Not reported | |
| **Ananthakrishnan *et al* (2016, United States)** | Women | 0.36 (0.18-0.75) | Not reported | | Not reported | | Not reported | Not reported | |
| | Men | 0.35 (0.21-0.59) | | | | | | | |
| **Liu *et al* (2016, Taiwan)** | Both sexes | 0.52 (0.44-0.62) | 40-64 years | 0.47 (0.36-0.62) | Not reported | | Not reported | Lipophilicity | 0.64 (0.53-0.77) |
| | | | | | | | | Hydrophilicity | 0.48 (0.35-0.66) |
| | | | | | | | | Simvastatin | 0.64 (0.47-0.87) |
| | | | | | | | | Lovastatin | 1.02 (0.75-1.38) |
| | | | 65-74 years | 0.54 (0.41-0.72) | | | | Atorvastatin | 0.65 (0.52-0.83) |
| | | | | | | | | Fluvastatin | 0.93 (0.63-1.35) |
| | | | ≥ 75 years | 0.48 (0.31-0.74) | | | | Pravastatin | 0.89 (0.60-1.32) |
| | | | | | | | | Rosuvastatin | 0.28 (0.17-0.45) |
| | Women | 0.55 (0.42-0.72) | | | | | | | |
| | Men | 0.49 (0.39-0.63) | | | | | | | |
| **Lee *et al* (2019, Korea)** | Women | 0.64 (0.46-0.90) | Not reported | | Not reported | | Not reported | Not reported | |
| | Men | 0.56 (0.42-0.75) | | | | | | | |
| **Yeh *et al* (2021, Taiwan)** | Women | 0.87 (0.69-1.11) | ≤ 49 years | 0.65 (0.34-1.25) | Not reported | | Not reported | Not reported | |

| | | | | | | | | |
|---|---|---|---|---|---|---|---|---|
| | Men | 0.70 (0.56-0.87) | ≥ 50 years | 0.75 (0.64-0.89) | | | | |
| **Author (year, country)** | **Sex categories** | | **Age categories** | | **Duration categories** | | **Intensity categories** | **Lipophilicity/ Active principle categories** |
| **Han *et al* (2021, Korea)** | Not reported | | Not reported | | Not reported | | Not reported | Not reported |
| **Erkinantti *et al* (2021, Finland)** | Not reported | | Not reported | | Not reported | | Not reported | Not reported |
| **Zhang *et al* (2022, United States)** | Not reported | | Not reported | | 1-5 years | 1.03 (0.92-1.16) | Not reported | Not reported |
| | | | | | 6-10 years | 1.05 (0.91-1.20) | | |
| | | | | | 11-15 years | 1.03 (0.80-1.32) | | |
| | | | | | > 15 years | 1.43 (1.04-1.99) | | |

**List of references**


1. Blais L, Desgagné A, LeLorier J. 3-Hydroxy-3-methylglutaryl coenzyme A reductase inhibitors and the risk of cancer: a nested case-control study. Arch Intern Med. 2000;160(15):2363-8. doi: 10.1001/archinte.160.15.2363.
2. Graaf MR, Beiderbeck AB, Egberts AC, Richel DJ, Guchelaar HJ. The risk of cancer in users of statins. J Clin Oncol. 2004;22(12):2388-94. doi: 10.1200/JCO.2004.02.027.
3. Kaye JA, Jick H. Statin use and cancer risk in the General Practice Research Database. Br J Cancer. 2004;90(3):635-7. doi: 10.1038/sj.bjc.6601566.
4. Khurana V, Jaganmohan S, Chalasani R, Singh T, Roy P, Caldito G, et al. Statins do not reduce colon cancer risk in humans: a case control study in half a million veterans: 746. Am J Gastroenterol. 2004; 99: S242.
5. Poynter JN, Gruber SB, Higgins PD, Almog R, Bonner JD, Rennert HS, et al. Statins and the risk of colorectal cancer. N Engl J Med. 2005;352(21):2184-92. doi: 10.1056/NEJMoa043792.
6. Rubin DT, Blumentals WA, Sheer RL, Steinbuch M, Law L. Statins and risk of colorectal cancer: Results from a large case-control study. Am J Gastroenterol 2005; 100: S394.
7. [a]Coogan PF, Rosenberg L, Brian LS. Statin Use and the Risk of 10 Cancers. Epidemiology. 2007; 213–219. DOI: 10.1097/01.ede.0000254694.03027.a1.
8. [b]Coogan PF, Smith J, Rosenberg L. Statin use and risk of colorectal cancer. J Natl Cancer Inst. 2007;99(1):32-40. doi: 10.1093/jnci/djk003.
9. Hoffmeister M, Chang-Claude J, Brenner H. Individual and joint use of statins and low-dose aspirin and risk of colorectal cancer: a population-based case-control study. Int J Cancer. 2007;121(6):1325-30. doi: 10.1002/ijc.22796.
10. Vinogradova Y, Hippisley-Cox J, Coupland C, Logan RF. Risk of colorectal cancer in patients prescribed statins, nonsteroidal anti-inflammatory drugs, and cyclooxygenase-2 inhibitors: nested case-control study. Gastroenterology. 2007;133(2):393-402. doi: 10.1053/j.gastro.2007.05.023.
11. Boudreau DM, Koehler E, Rulyak SJ, Haneuse S, Harrison R, Mandelson MT. Cardiovascular medication use and risk for colorectal cancer. Cancer Epidemiol Biomarkers Prev. 2008;17(11):3076-80. doi: 10.1158/1055-9965.EPI-08-0095.
12. Yang YX, Hennessy S, Propert K, Hwang WT, Sarkar M, Lewis JD. Chronic statin therapy and the risk of colorectal cancer. Pharmacoepidemiol Drug Saf. 2008;17(9):869-76. doi: 10.1002/pds.1599.
13. Hachem C, Morgan R, Johnson M, Kuebeler M, El-Serag H. Statins and the risk of colorectal carcinoma: a nested case-control study in veterans with diabetes. Am J Gastroenterol. 2009;104(5):1241-8. doi: 10.1038/ajg.2009.64

14. Shadman M, Newcomb PA, Hampton JM, Wernli KJ, Trentham-Dietz A. Non-steroidal anti-inflammatory drugs and statins in relation to colorectal cancer risk. World J Gastroenterol. 2009;15(19):2336-9. doi: 10.3748/wjg.15.2336.
15. Robertson DJ, Riis AH, Friis S, Pedersen L, Baron JA, Sørensen HT. Neither long-term statin use nor atherosclerotic disease is associated with risk of colorectal cancer. Clin Gastroenterol Hepatol. 2010;8(12):1056-61. doi: 10.1016/j.cgh.2010.08.010
16. Cheng MH, Chiu HF, Ho SC, Tsai SS, Wu TN, Yang CY. Statin use and the risk of colorectal cancer: a population-based case-control study. World J Gastroenterol. 2011;17(47):5197-202. doi: 10.3748/wjg.v17.i47.5197.
17. Samadder NJ, Mukherjee B, Huang SC, Ahn J, Rennert HS, Greenson JK, et al. Risk of colorectal cancer in self-reported inflammatory bowel disease and modification of risk by statin and NSAID use. Cancer. 2011;117(8):1640-8. doi: 10.1002/cncr.25731.
18. Vinogradova Y, Coupland C, Hippisley-Cox J. Exposure to statins and risk of common cancers: a series of nested case-control studies. BMC Cancer. 2011;11:409. doi: 10.1186/1471-2407-11-409.
19. Broughton T, Sington J, Beales IL. Statin use is associated with a reduced incidence of colorectal cancer: a colonoscopy-controlled case-control study. BMC Gastroenterol. 2012;12:36. doi: 10.1186/1471-230X-12-36.
20. Lakha F, Theodoratou E, Farrington SM, Tenesa A, Cetnarskyj R, Din FV, et al. Statin use and association with colorectal cancer survival and risk: case control study with prescription data linkage. BMC Cancer. 2012;12:487. doi: 10.1186/1471-2407-12-487.
21. Deshpande G. Association between cardiovascular drugs and colon cancer [Internet]. Available from: URL: http://archive.hshsl.umaryland.edu/handle/10713/2752. Accesed Sep 15 2021
22. Björkhem-Bergman L, Backheden M, Söderberg Löfdal K. Statin treatment reduces the risk of hepatocellular carcinoma but not colon cancer-results from a nationwide case-control study in Sweden. Pharmacoepidemiol Drug Saf. 2014;23(10):1101-6. doi: 10.1002/pds.3685.
23. Sehdev A, Shih YC, Huo D, Vekhter B, Lyttle C, Polite B. The role of statins for primary prevention in non-elderly colorectal cancer patients. Anticancer Res. 2014;34(9):5043-50. PMID: 25202089.
24. Mamtani R, Lewis JD, Scott FI, Ahmad T, Goldberg DS, Datta J, et al. Disentangling the Association between Statins, Cholesterol, and Colorectal Cancer: A Nested Case-Control Study. PLoS Med. 2016;13(4):e1002007. doi: 10.1371/journal.pmed.1002007.
25. Horibe Y, Adachi S, Ohno T, Goto N, Okuno M, Iwama M, et al. Alpha-glucosidase inhibitor use is associated with decreased colorectal neoplasia risk in patients with type 2 diabetes mellitus receiving colonoscopy: a retrospective study. Oncotarget. 2017;8(58):97862-97870. doi: 10.18632/oncotarget.18416.
26. Renman D, Lundberg E, Gunnarsson U, Strigård K. Statin consumption as a risk factor for developing colorectal cancer: a retrospective case study. World J Surg Oncol. 2017;15(1):222. doi: 10.1186/s12957-017-1287-0.

27. Ibáñez-Sanz G, Guinó E, Pontes C, Quijada-Manuitt MÁ, de la Peña-Negro LC, Aragón M, et al. Statin use and the risk of colorectal cancer in a population-based electronic health records study. Sci Rep. 2019;9(1):13560. doi: 10.1038/s41598-019-49877-5.
28. Dickerman BA, García-Albéniz X, Logan RW, Denaxas S, Hernán MA. Emulating a target trial in case-control designs: an application to statins and colorectal cancer. Int J Epidemiol. 2020;49(5):1637-1646. doi: 10.1093/ije/dyaa144.
29. Friis S, Poulsen AH, Johnsen SP, McLaughlin JK, Fryzek JP, Dalton SO, et al. Cancer risk among statin users: a population-based cohort study. Int J Cancer. 2005;114(4):643-7. doi: 10.1002/ijc.20758.
30. Jacobs EJ, Rodriguez C, Brady KA, Connell CJ, Thun MJ, Calle EE. Cholesterol-lowering drugs and colorectal cancer incidence in a large United States cohort. J Natl Cancer Inst. 2006;98(1):69-72. doi: 10.1093/jnci/djj006.
31. Setoguchi S, Glynn RJ, Avorn J, Mogun H, Schneeweiss S. Statins and the risk of lung, breast, and colorectal cancer in the elderly. Circulation. 2007;115(1):27-33. doi: 10.1161/CIRCULATIONAHA.106.650176.
32. Farwell WR, Scranton RE, Lawler EV, Lew RA, Brophy MT, Fiore LD, et al. The association between statins and cancer incidence in a veterans population. J Natl Cancer Inst. 2008;100(2):134-9. doi: 10.1093/jnci/djm286.
33. Friedman GD, Flick ED, Udaltsova N, Chan J, Quesenberry CP Jr, Habel LA. Screening statins for possible carcinogenic risk: up to 9 years of follow-up of 361,859 recipients. Pharmacoepidemiol Drug Saf. 2008;17(1):27-36. doi: 10.1002/pds.1507. Erratum in: Pharmacoepidemiol Drug Saf. 2008 Jul;17(7):751.
34. Flick ED, Habel LA, Chan KA, Haque R, Quinn VP, Van Den Eeden SK, et al. Statin use and risk of colorectal cancer in a cohort of middle-aged men in the US: a prospective cohort study. Drugs. 2009;69(11):1445-57. doi: 10.2165/00003495-200969110-00004.
35. Singh H, Mahmud SM, Turner D, Xue L, Demers AA, Bernstein CN. Long-term use of statins and risk of colorectal cancer: a population-based study. Am J Gastroenterol. 2009;104(12):3015-23. doi: 10.1038/ajg.2009.574.
36. Haukka J, Sankila R, Klaukka T, Lonnqvist J, Niskanen L, Tanskanen A, et al. Incidence of cancer and statin usage--record linkage study. Int J Cancer. 2010;126(1):279-84. doi: 10.1002/ijc.24536.
37. Jacobs EJ, Newton CC, Thun MJ, Gapstur SM. Long-term use of cholesterol-lowering drugs and cancer incidence in a large United States cohort. Cancer Res. 2011;71(5):1763-71. doi: 10.1158/0008-5472.CAN-10-2953.
38. Lee JE, Baba Y, Ng K, Giovannucci E, Fuchs CS, Ogino S, et al. Statin use and colorectal cancer risk according to molecular subtypes in two large prospective cohort studies. Cancer Prev Res (Phila). 2011;4(11):1808-15. doi: 10.1158/1940-6207.CAPR-11-0113.
39. Clancy Z, Keith SW, Rabinowitz C, Ceccarelli M, Gagne JJ, Maio V. Statins and colorectal cancer risk: a longitudinal study. Cancer Causes Control. 2013;24(4):777-82. doi: 10.1007/s10552-013-0160-x.

40. Leung HW, Chan AL, Lo D, Leung JH, Chen HL. Common cancer risk and statins: a population-based case-control study in a Chinese population. Expert Opin Drug Saf. 2013;12(1):19-27. doi: 10.1517/14740338.2013.744392.
41. Ananthakrishnan AN, Cagan A, Cai T, Gainer VS, Shaw SY, Churchill S, et al. Statin Use Is Associated With Reduced Risk of Colorectal Cancer in Patients With Inflammatory Bowel Diseases. Clin Gastroenterol Hepatol. 2016;14(7):973-9. doi: 10.1016/j.cgh.2016.02.017.
42. Liu JC, Hao WR, Hsu YP, Sung LC, Kao PF, Lin CF, et al. Statins dose-dependently exert a significant chemopreventive effect on colon cancer in patients with chronic obstructive pulmonary disease: A population-based cohort study. Oncotarget. 2016;7(40):65270-65283. doi: 10.18632/oncotarget.11263.
43. Lee JW, You NY, Kim Y, Kim Y, Kim J, Kang HT. Statin use and site-specific risk of colorectal cancer in individuals with hypercholesterolemia from the National Health Insurance Service-National Health Screening Cohort (NHIS-HEALS). Nutr Metab Cardiovasc Dis. 2019;29(7):701-709. doi: 10.1016/j.numecd.2019.04.002.
44. Zhang Y, Wu K, Chan AT, Meyerhardt JA, Giovannucci EL. Long-Term Statin Use, Total Cholesterol Level, and Risk of Colorectal Cancer: A Prospective Cohort Study. Am J Gastroenterol. 2022;117(1):158-166. doi: 10.14309/ajg.0000000000001543.
45. Yeh JJ, Lai JN, Lin CL, Hsu CY, Kao CH. Time-dependent propensity-matched general population study of the effects of statin use on cancer risk in an interstitial lung disease and pulmonary fibrosis cohort. BMJ Open. 2021;11(10):e047039. doi: 10.1136/bmjopen-2020-047039.
46. Han KT, Kim S. Do cholesterol levels and continuity of statin use affect colorectal cancer incidence in older adults under 75 years of age? PLoS One. 2021;16(4):e0250716. doi: 10.1371/journal.pone.0250716.
47. Erkinantti S, Marttila M, Sund R, Arffman M, Urpilainen E, Puistola U, et al. Association of Metformin, Other Antidiabetic Medications, and Statins With Incidence of Colon Cancer in Patients With Type 2 Diabetes. Clin Colorectal Cancer. 2021;20(2):e113-e119. doi: 10.1016/j.clcc.2020.11.003.

**Figure S3. Funnel plot**

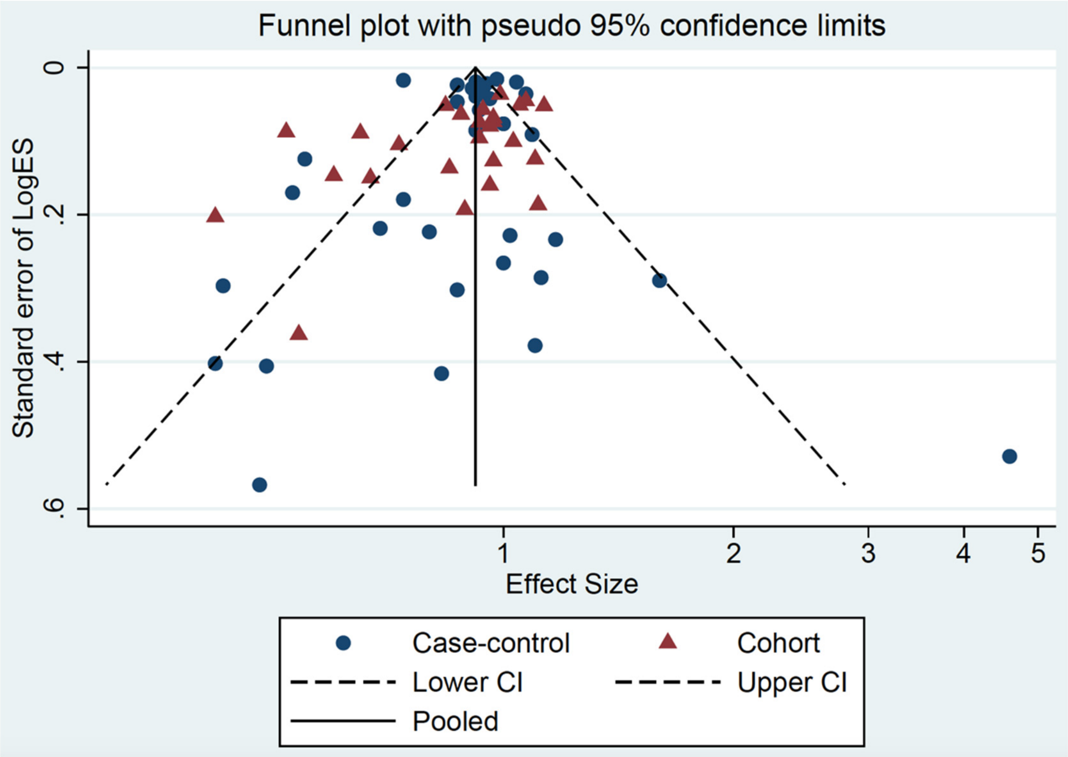


ES: standard error; CI: confidence interval
